# The potential of the multivariate multilevel model for analysing correlated multiple outcomes: a simulation study

**DOI:** 10.1186/1745-6215-16-S2-P151

**Published:** 2015-11-16

**Authors:** Victoria Vickerstaff, Gareth Ambler, Rumana Z Omar

**Affiliations:** 1 Department of Statistical Science, University College London, London, UK; 2 Marie Curie Palliative Care Research Department, University College London, London, UK

## 

In clinical trials, multiple primary outcomes are often needed to assess the effectiveness of an intervention as a single primary outcome can be insufficient to describe all aspects of a complex disorder. These outcomes are often correlated. Many procedures for addressing multiple outcomes have been introduced; however, most of the commonly used methods do not make use of the correlations among the outcomes. The multivariate multilevel model (MMM) can be used to analyse multiple primary outcomes. The MMM views multiple outcomes as repeated measures clustered within individuals, consequently, missing data is automatically taken into account through the correlations among the outcomes.

This simulation study assesses the type I error and power to detect true intervention effects when using the MMM compared to using a separate model for each outcome and performing appropriate adjustments to the p-values, including the Bonferroni, Holm, Hommel and Hochberg adjustments. Simulation scenarios include varying the number of outcomes, between outcome correlations, and the degree and pattern of missingness.

The simulation study shows that power gains can be obtained when using the MMM if the outcomes are correlated. Consequently, if the MMM is used, a smaller sample size may be required to obtain the required level of power. We are currently investigating the use of a combination of the MMM and another method to adjust the type I error to provide an optimal analysis. MMM shows potential, particularly when there is missing data (including missing not at random data).

